# The protective effects of oral low-dose quercetin on diabetic nephropathy in hypercholesterolemic mice

**DOI:** 10.3389/fphys.2015.00247

**Published:** 2015-09-02

**Authors:** Isabele B. S. Gomes, Marcella L. Porto, Maria C. L. F. S. Santos, Bianca P. Campagnaro, Agata L. Gava, Silvana S. Meyrelles, Thiago M. C. Pereira, Elisardo C. Vasquez

**Affiliations:** ^1^Laboratory of Translational Physiology, Health Sciences Center, Federal University of Espirito SantoVitoria, Brazil; ^2^Pharmaceutical Sciences Graduate Program, Vila Velha UniversityVila Velha, Brazil; ^3^Department of Biotechnology, Federal Institute of Education, Science and Technology (IFES)Vila Velha, Brazil; ^4^Emescam School of Health SciencesVitoria, Brazil

**Keywords:** quercetin, apoE, diabetes, streptozotocin, atherosclerosis, nephropathy

## Abstract

**Aims:** Diabetic nephropathy (DN) is one of the most important causes of chronic renal disease, and the incidence of DN is increasing worldwide. Considering our previous report (Gomes et al., 2014) indicating that chronic treatment with oral low-dose quercetin (10 mg/Kg) demonstrated anti-oxidative, anti-apoptotic and renoprotective effects in the C57BL/6J model of DN, we investigated whether this flavonoid could also have beneficial effects in concurrent DN and spontaneous atherosclerosis using the apolipoprotein E-deficient mouse (apoE^−/−^).

**Methods:** Streptozotocin was used to induce diabetes (100 mg/kg/day, 3 days) in male apoE^−/−^ mice (8 week-old). After 6 weeks, the mice were randomly separated into DQ: diabetic apoE^−/−^ mice treated with quercetin (10 mg/kg/day, 4 weeks, *n* = 8), DV: diabetic ApoE^−/−^ mice treated with vehicle (*n* = 8) and ND: non-treated non-diabetic mice (*n* = 8).

**Results:** Quercetin treatment diminished polyuria (~30%; *p* < 0.05), glycemia (~25%, *p* < 0.05), normalized the hypertriglyceridemia. Moreover, this bioflavonoid diminished creatininemia (~30%, *p* < 0.01) and reduced proteinuria but not to normal levels. We also observed protective effects on the renal structural changes, including normalization of the index of glomerulosclerosis and kidney weight/body weight.

**Conclusions:** Our data revealed that quercetin treatment significantly reduced DN in hypercholesterolemic mice by inducing biochemical changes (decrease in glucose and triglycerides serum levels) and reduction of glomerulosclerosis. Thus, this study highlights the relevance of quercetin as an alternative therapeutic option for DN, including in diabetes associated with dyslipidemia.

## Introduction

Diabetic nephropathy (DN) is the major cause of chronic renal disease in industrialized nations and is linked with a significant increase in cardiovascular morbi-mortality (Foggensteiner et al., 2001; Ahmad, 2015; Donate-Correa et al., 2015). It occurs because of an interaction between both genetic and environmental factors in diabetic individuals, such as genetic pre-disposition, sedentary lifestyle, hypertension, persistent hyperglycemia and dyslipidemia (Lassila et al., 2004; Matheus et al., 2013; Ahmad, 2015). By different routes, all these factors can contribute directly and/or indirectly to an abnormal balance between reactive oxygen species (ROS) production and its antioxidant mechanisms aggravating the pathogenesis of DN (Lassila et al., 2004; Xu et al., 2006; Duran-Salgado and Rubio-Guerra, 2014; Gorin and Wauquier, 2015; Lv et al., 2015).

In recent decades, although the use of animal models has provided new insights into understanding the pathogenesis, diagnosis and treatment of nephropathy (Balakumar et al., 2008), most of the models employed do not associate comorbidities, limiting the extrapolation of these studies to humans. In an attempt to combine the effects of two severe clinical risk factors (dyslipidemia and diabetes) for renal disease, we used the hyperlipidemic diabetic apolipoprotein E-deficient (apoE^−/−^) mouse in our study. Recent data indicate that when this animal is administered streptozotocin (STZ), a toxin widely used to induce experimental diabetes (Like and Rossini, 1976; Vessal et al., 2003), it develops accelerated hypercholesterolemia/atherosclerosis (Candido et al., 2004; Vedantham et al., 2011) and nephropathy (Wen et al., 2002; Lassila et al., 2004; Xu et al., 2006).

Considering that only partial renoprotection from DN is achieved by current standard therapies (e.g., by the inhibition of the renin-angiotensin-aldosterone system), the search for alternative, effective and safer therapeutic approaches is an interesting goal. In this context, recent findings from our laboratory (Gomes et al., 2014) demonstrate that an orally administered low-dose of the antioxidant quercetin (10 mg/Kg), a bioflavonoid ubiquitously contained in vegetables and fruits (Kawabata et al., 2015), exhibits metabolic, anti-oxidative, anti-apoptotic and renoprotective effects in the C57BL/6J mouse model of DN. In parallel, others have found cardiovascular protection from quercetin in the ApoE^−/−^ mouse model (Lara-Guzman et al., 2012; Ulasova et al., 2013). In light of these evidences, we tested the hypothesis that, due to its antioxidant properties, quercetin treatment could improve metabolic parameters and renal function in the diabetic apoE^−/−^ mouse model.

## Materials and methods

### Animals

The apoE^−/−^ male mice (8 week-old, *n* = 24) were obtained from the animal facilities of the Laboratory of Translational Physiology, at the Federal University of Espirito Santo, Brazil. The mice were fed a normal laboratory chow diet (Labina®) and water *ad libitum* until the time of the experiments. The animals were housed at 22°C, 50% humidity with a 12 h-light/12 h-dark cycle. All of the procedures were conducted in accordance with of the institutional guidelines for animal research, and the protocols were previously certified by the Institutional Ethics Committee for Use of Animals (Protocol # 013/2010).

### Experimental protocol

Diabetes was induced by three daily intraperitoneal injections of streptozotocin (STZ, Boehringer Mannheim, Mannheim, Germany) at a dose of 100 mg/kg diluted in citrate buffer solution (10 mM, pH 4.5). Non-diabetic apoE^−/−^ mice were administered the vehicle citrate buffer and served as controls. One week after the STZ injection, the glycemia was measured using blood samples (tail vein) obtained from mice after 6 h of inanition. The inclusion criteria were those animals that 1 week after STZ injection exhibited hyperglycemia (>250 mg/dL), when it was confirmed at least in two independent moments (success rate was approximately 65%). After 6 weeks, the animals were randomized to receive vehicle (soy oil, DV, *n* = 8) or oral quercetin (DQ, *n* = 8; Sigma, St. Louis, MO, USA) at a dosage of 10 mg/kg per day orally for 4 weeks, based on our prior study (Gomes et al., 2014) and others (Ajay et al., 2006; Machha and Mustafa, 2005).

### Metabolic and biochemical parameters

The body weight of all the animals was measured weekly. At week 4, the mice were adapted to 24-h in individual metabolic cages. Thereafter, a known quantity of food and water were positioned in the feeder and the drinking bottles, respectively. After 24 h, we measured the volume of water and amount of chow remaining in the cages. Urine volume was measured and protein concentration was determined by the Bradford method (Bradford, 1976). Finally, animals received a lethal dose of thiopental (Cristalia, Sao Paulo, Brazil, 200 mg/kg, i.p.) after 6 h of inanition in the morning. The blood samples were collected using the retro-orbital sinus of the mouse as a source of venous blood for all measurements, with exception of the determination of glycemia, which was trough the tail venipuncture. The biochemical analysis of glucose, triglycerides, cholesterol, creatinine, urea and uric acid measurements were performed by colorimetric kits. Animals were perfused with cold PBS (pH 7.4, 0.1 mol/L) through the left ventricle. Creatinine clearance was calculated using serum and urine creatinine levels and urine flow through the standardized formula: [urine creatinine concentration (mg/dL) × 24 h urine volume (μL)]/[serum creatinine concentration (mg/dL) 1440 min].

### Kidney histology

After perfusion of the animal, the kidneys were carefully fixed with Duboscq solution (aqueous solution of 0.4% picric acid, 54% ethanol, 27% formaldehyde, and 7% acetic acid), weighed and managed for histological and morphometric analysis. The samples were dehydrated in increasing concentrations of alcohol and finally mounted in paraffin blocks. Thereafter, the kidneys were sliced using a microtome into 3-μm-thick cross-sections with hematoxylin-eosin staining. Images were obtained with video camera (VKC150, Hitachi, Tokyo, Japan) connected to a microscope (AX70, Olympus, Center Valley, PA, USA). The mean glomerular tuft area of each kidney was obtained by calculating the mean value of 30 individual glomeruli measured by Image J software (version 1.33u, Public Domain). Masson's trichrome staining was used to quantify glomerulosclerosis. A total of 30 glomeruli were used to calculate the percentage of the stained area for each kidney using the Image J program (Public Domain Image Processing Program, National Institutes of Health, Bethesda, MD).

### Statistical analysis

The data are presented as the mean ± SEM. The normality of the variables was tested by Kolmogorov-Smirnov. The statistical analysis was performed using One-Way analysis of variance (ANOVA) followed by the Tukey's *post*-*hoc* test using Prism software (Prism 6, GraphPad Software, Inc., San Diego, CA, USA). The level of significance was set at *p* < 0.05.

## Results

### The effects of quercetin on metabolic parameters

**Figure 1:**
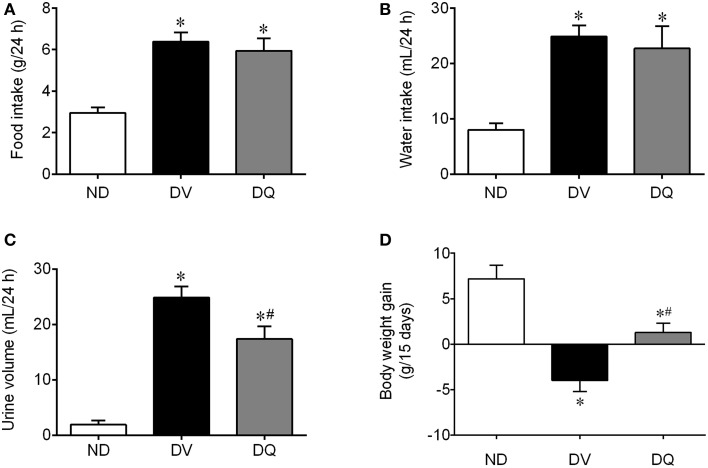
**Food and water intake, urine volume and body weight gain in diabetic apoE^−/−^ mice treated with quercetin (DQ) compared with diabetic apoE^−/−^ mice administered vehicle (DV) compared with non-diabetic apoE^−/−^ (ND) mice**. Values are the means ± SEM for *n* = 6–8 mice per group. ^*^p < 0.05 vs. ND, ^#^*p* < 0.05 vs. DV.

Figure 1 summarizes the data obtained through metabolic cages (food intake, water intake, and urine volume) and the body weight gain in the three groups studied. DV mice showed hyperphagia (*p* < 0.05, Figure 1A) and polydipsia (*p* < 0.05, Figure 1B) when compared with ND mice and no effect of quercetin was observed on these parameters. Interestingly, DV mice showed polyuria (*p* < 0.05), which was reduced by approximately 30% of DQ mice (*p* < 0.05) (Figure 1C). Body weight was statistically similar in the three groups at the beginning of the protocol, but as shown in Figure 1D, over the 2-week period, only the DV mice showed reductions in body weight, in contrast to the ND mice and DQ mice (*p* < 0.05), which showed significant increases in body weight.

### Effects of quercetin on biochemical parameters

**Figure 2:**
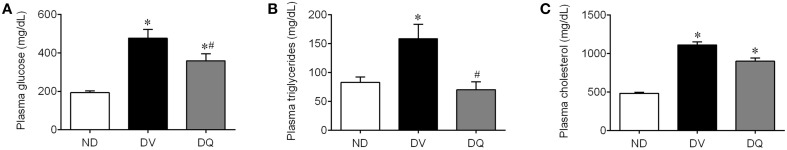
**Total plasma glucose (A), triglycerides (B), and cholesterol (C) in diabetic apoE^−/−^ mice treated with quercetin (DQ) compared with diabetic apoE^−/−^ mice administered vehicle (DV) compared with non-diabetic apoE^−/−^ (ND) mice**. Values are the means ± SEM for *n* = 6–8 mice per group. ^*^p < 0.05 vs. ND, ^#^*p* < 0.05 vs. DV.

As summarized in Figure 2, DV mice exhibited a significant augmentation in glycemia (2.5-fold), triglycerides (1.9-fold) and total cholesterol (2.3-fold) when compared with control ND mice (*p* < 0.05). The treatment of diabetic apoE^−/−^ mice with quercetin caused significant attenuation of plasma glucose (~25%) and abolished the hypertriglyceridemia (*p* < 0.05); however, this dose of quercetin did not reverse the hypercholesterolemia.

### Effects of quercetin on kidney functional and morphometric parameters

**Figure 3:**
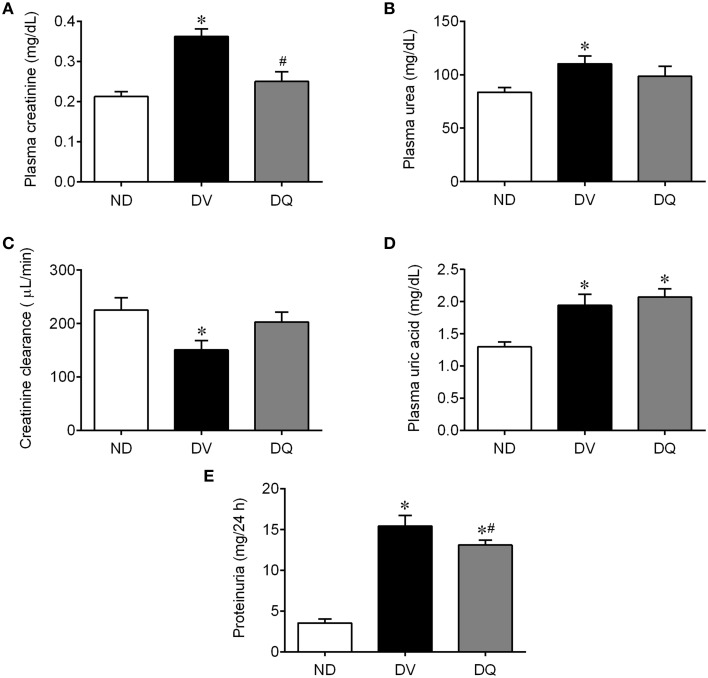
**Plasma creatinine (A), urea (B), creatinine clearance (C), uric acid (D), and proteinuria (E) in diabetic apoE^−/−^ mice treated with quercetin (DQ) compared with diabetic apoE^−/−^ mice administered vehicle (DV) compared with non-diabetic apoE^−/−^ (ND) mice**. Values are the means ± SEM for *n* = 6–8 mice per group. ^*^p < 0.05 vs. ND, ^#^*p* < 0.05 vs. DV.

Figure 3 shows the mean values of the traditional renal function biomarkers. As expected, DV mice exhibited significantly high plasma concentrations of creatinine (Figure 3A), urea (Figure 3B), uric acid (Figure 3D), and impairment of renal clearance (Figure 3C) compared with ND animals (*p* < 0.05). In DQ mice, plasma creatinine and clearance returned to baseline levels (*p* < 0.05, Figure 3C). In addition, quercetin did not modify the high plasma both urea and uric acid (*p* > 0.05). Proteinuria was significantly increased (4.4-fold, *p* < 0.05) in the DV mice compared to the ND mice (*p* < 0.01, Figure 3E). Treatment with quercetin showed a tendency to reduce proteinuria (~15%), but the levels were still significantly higher than those of the ND mice.

**Figure 4:**
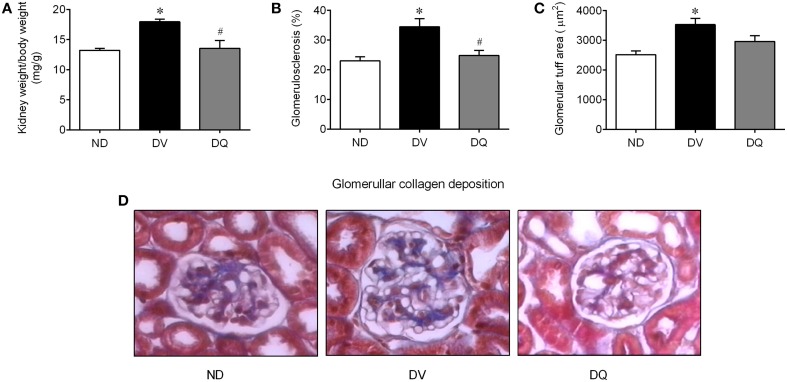
**Kidney weight (wt)/body weight ratio (A), glomerulosclerosis (B), glomerular tuff area (C) in diabetic apoE^−/−^ mice treated with quercetin (DQ) compared with diabetic apoE^−/−^ mice administered vehicle (DV) compared with non-diabetic apoE^−/−^ (ND) mice**. Photomicrographs **(D)** are representative glomerular sections (magnification of 400x), stained with Masson's trichrome. Values are the means ± SEM for *n* = 6–8 mice per group. ^*^p < 0.05 vs. ND, ^#^*p* < 0.05 vs. DV.

Diabetes was related to an augment of ~35% in the kidney weight/body weight ratio when compared with ND mice (*p* < 0.05), whereas quercetin reversed this consequence of diabetes in the apoE^−/−^ mice (*p* < 0.05, Figure 4A). As illustrated in the typical microscopy images (Figure 4D), the glomerulosclerosis, which was characterized by glomerular hyperplasia and by deposition of extracellular matrix in the mesangium, was more prominent in the DV mice, than in the ND mice, and quercetin showed a favorable effect on this condition. More specifically, the analysis of glomerulosclerosis demonstrated a significant increase of approximately 50% when compared with ND mice (*p* < 0.05), and quercetin abolished this glomerular injury (Figure 4B). Additionally, the mean glomerular tuft area of each kidney revealed an increase of approximately 40% compared to those of ND mice (*p* < 0.05), and DQ had a tendency to attenuate this glomerular injury (Figure 4C).

## Discussion

Recent data from our laboratory showed that oral low-dose quercetin ameliorated the consequences of hyperglycemia-induced ROS overproduction in the kidney in wild-type diabetic mice (Gomes et al., 2014), the most common genetic background for gene-modified mice (Haug et al., 2013). The novelty of this study is that the same dose of this bioflavonoid was capable of reducing the classical signs of diabetes and attenuated the progression of nephropathy in diabetic-induced apoE^−/−^ mice. These data are reinforced by a reduction in hyperglycemia, hypertriglyceridemia, azotemia, proteinuria and the diminution of mesangial matrix expansion in the kidneys of diabetic apoE^−/−^ mice.

Although there are limitations to the experimental diabetic mouse model compared to humans (Wu and Huan, 2007; Müller et al., 2012), STZ-induced diabetic ApoE^−/−^ mouse is an interesting model for exploring new therapeutic options for diabetes-associated dyslipidemia and renal injury. First, the diabetic condition in this model is preserved for many weeks, which allowed us long periods of treatment with quercetin. Second, the diabetic status is not refractory to medical interventions (Wu and Huan, 2007), which was evidenced in our study in the antidiabetic protection observed with administration of this bioflavonoid. Moreover, in order to avoid interference with the direct nephrotoxic effects of STZ, the experiments were performed after 6 weeks to avoid acute renal injury bias (Ortega et al., 2013; Gomes et al., 2014).

As in other STZ models, STZ-induced diabetic ApoE^−/−^ mice showed damaged β cells that compromised the secretory capacity of insulin (Like and Rossini, 1976; Wu and Yan, 2015). Consequently, this atherosclerotic model exhibits the expected progressive signs of the disease, such as hyperglycemia, polyuria, polydipsia, polyphagia, proteinuria and the decline of renal function, similar to those in C57BL/6J mice (Gomes et al., 2014). Based on the 5 stages of the clinical classification of DN and by the sum of these characteristics (Mogensen et al., 1983; Jerums et al., 2009), we consider this model to correspond to stage 4 clinical classification because the animals exhibited a diminished creatinine clearance and proteinuria similar to that observed in C57BL/6J mice (Gomes et al., 2014). Exceptionally, only the loss of body weight was more evident when compared to their respective genetic background, probably due to the lack of apoE. Pendse et al. (2009) demonstrated that the absence of this apolipoprotein contributes directly to the suppression of body weight gain and consequent fat accumulation in apoE^−/−^ mice, which corroborates our hypothesis.

For more than almost 20 years, it has been known that oxidative stress plays a crucial role in the development of diabetic complications (Baynes, 1991; Wright et al., 2006; Alam et al., 2014). In this context, the search for nontoxic natural antioxidant compounds to prevent oxidative damage in experimental models of diabetes (Wang et al., 2012) and in diabetic patients (Valensi et al., 2005; Lobo et al., 2010; Sunarwidhi et al., 2014) has been intensified in recent years. Typically, the best candidates are molecules that exhibit high antioxidant activity, long half-lives (Sesink et al., 2001; Manach et al., 2005), and high mitochondrial permeability (Ortega and García, 2009) and are able to suppress pro-oxidant enzymes and stimulate antioxidant enzymes (Bouayed and Bohn, 2010). Interestingly, quercetin exhibits all of these advantages (Sanders et al., 2001; Ortega and García, 2009; Gomes et al., 2014). Similarly, recent studies from our group (Gomes et al., 2014) and other groups (Pereira Braga et al., 2013) have demonstrated that this bioflavonoid diminishes ROS bioavailability through distinct pathways: (1) by the chelation of metals, (2) by neutralizing lipid peroxyl radicals, (3) by interacting directly with O^−^_2_ during initiation and (4) by increasing the activity of glutathione peroxidase/reductase/transferase, superoxide dismutase and catalase (Oršolic et al., 2011; Alam et al., 2014).

Although the antioxidative benefits of quercetin are well-established in diabetic experimental models (Kobori et al., 2009; Oršolic et al., 2011; Kanter et al., 2012), other effects still require further investigation (Youl et al., 2010; Gomes et al., 2014). Interestingly, our results demonstrated for the first time that quercetin attenuates hyperglycemia in a mouse model of dyslipidemia and diabetes, as observed recently by others in diabetic rats (Kanter et al., 2012), in Balb/C mice (Kobori et al., 2009) and by us in diabetic C57BL/6J mice (Gomes et al., 2014). The beneficial effect of quercetin on glycemia may work through different mechanisms, such as through the stimulation of glucose influx via GLUT4 (Alam et al., 2014; Xu et al., 2014) and via augmented glucokinase activity and, consequently, the increase in glucose liver uptake, inhibiting hepatic glycogenolysis and gluconeogenesis (Alam et al., 2014). Moreover, it has been shown that quercetin can inhibit α-glucosidase (Ishikawa et al., 2007; Kim et al., 2011) and the intestinal glucose transporter GLUT2 (Kwon et al., 2007), reducing the absorption of monosaccharides in the small intestine. Because the low dose of quercetin we used has been associated with intrinsic low bioavailability, interference with the absorption of monosaccharides seems reasonable (Gomes et al., 2014) and is consistent with the findings of Galindo et al. (2012), who showed a better effect when compared to administration via the intraperitoneal route. However, we cannot exclude the protective role of quercetin in Langerhans β-cells from damage on improving insulin production in STZ models, as observed by others (Vessal et al., 2003; Kim et al., 2011). Independent of this mechanism, the attenuation of chronic hyperglycemia reduces damage to a number of cell types through several pathways, such as the augmented formation of advanced glycation end-products (AGEs) and its respective receptor, polyol pathway flux, the overactivity of the hexosamine pathway, activation of protein kinase C (PKC) isoforms and even mitochondrial dysfunction (Wright et al., 2006; Giacco and Brownlee, 2010; Alam et al., 2014), which attenuates progressive damage to major target organs.

Although treatment with quercetin ameliorated the reduction in body weight gain and polyuria, it probably prevented reductions in body weight gain and polyuria. This effect may be justified as a consequence of better glycemic control, with a reduction of the compensatory lipolytic response and consequent normalization of triglyceridemia without modifying the hypercholesterolemia, as recently observed by our group (Gomes et al., 2014) and others (Ozcelik et al., 2011). Furthermore, we cannot reject the possibility of a modification in the non-HDL/HDL ratio, which maintains invariable total serum cholesterol levels (Negi et al., 2013; Gomes et al., 2014).

In a previous study, we have shown that apoE^−/−^ mice exhibit early impaired renal function when compared with normocholesterolemic C57 mice (Balarini et al., 2011). Now, using the experimental model of DN aggravated by hyperlipidemia, we observed signs of renal glomerular injury, which could be justified by azotemia with reduced creatinine clearance associated with the histological assessment. Moreover, the glomerular tuft size was exacerbated in diabetic apoE^−/−^ mice, indicating an initial diabetes-induced renal injury, which is consistent with the literature (Xu et al., 2006; Menini et al., 2015). For the first time, our study demonstrates that treatment with quercetin ameliorated the glomerulosclerosis and recovered the kidney weight/body weight ratio. However, we emphasize that this latter finding should not be interpreted as an occurrence of renal hypertrophy because we observed that the diabetic animals exhibited lower body weight. Additionally, this bioflavonoid also exhibited marked beneficial effects on renal function as indicated by the significant decrease of creatininemia, restoration of the clearance of creatinine and tended to reduce the proteinuria in diabetic apoE^−/−^ mice. The non-modification of the uremia and uric acid parameters may be justified by the following: (1) an intense purine and amino acid catabolism (respectively) in this induced diabetic experimental model (Gomes et al., 2014) and (2) by more glomerular sensitivity to oxidative injuries than other nephron segments (Schena and Gesualdo, 2005; Gomes et al., 2014), favoring the amelioration of renal filtration that we observed in the present study. All of these renoprotective effects of quercetin could be explained by direct benefits such as the vasorelaxant effect in vascular tissues recently described (Schena and Gesualdo, 2005; Lodi et al., 2009; Galindo et al., 2012), in addition to indirect effects such as its hypoglycemic/anti-dyslipidemic actions (Lassila et al., 2004) and the reduction of ROS formation (Gomes et al., 2014). Likewise, we cannot reject that quercetin can also positively modulate the functional activities of endothelial progenitor cells (EPCs) in vascular and kidney repair after damage, as observed recently *in vitro* by Zhao et al. (2014), offering new insights into antidiabetic therapies.

In conclusion, we have demonstrated that an oral administered low-dose of quercetin exhibits antidiabetic and renoprotective effects in a mouse model of concurrent apoE^−/−^-induced hypercholesterolemia and STZ-induced DN. Although further studies are needed to reveal the intrinsic mechanisms involved, this bioflavonoid is a potential nutraceutical alternative to prevent and/or treat renal dysfunction caused by diabetes and dyslipidemia as shown in the present study.

## Author contributions

Conception and design of the experiments: IG, MP, AG, and EV. Collection, analysis and interpretation of the data: IG, MS, BC, EV, and TP. Drafting or revising the article critically for intellectual content: SM, TP, and EV.

### Conflict of interest statement

The authors declare that the research was conducted in the absence of any commercial or financial relationships that could be construed as a potential conflict of interest.

## References

[B1] Ahmad J. (2015). Management of diabetic nephropathy: recent progress and future perspective. Diabetes Metab. Syndr. S1871-4021(15)00021-1. [Epub ahead of print]. 10.1016/j.dsx.2015.02.008 25845297

[B2] Ajay M. Achike F. I. Mustafa A. M. Mustafa M. R. (2006). Effect of quercetin on altered vascular reactivity in aortas isolated from streptozotocin-induced diabetic rats. Diabetes Res. Clin. Pract. 73, 1–7. 10.1016/j.diabres.2005.11.004 16378655

[B3] Alam M. M. Meerza D. Naseem I. (2014). Protective effect of quercetin on hyperglycemia, oxidative stress and DNA damage in alloxan induced type 2 diabetic mice. Life Sci. 109, 8–14. 10.1016/j.lfs.2014.06.005 24946265

[B4] Balakumar P. Chakkarwar V. A. Kumar V. Jain A. Reddy J. Singh M. (2008). Experimental models for nephropathy. J. Renin Angiotensin Aldosterone Syst. 9, 189–195. 10.1177/1470320308098343 19126658

[B5] Balarini C. M. Oliveira M. Z. Pereira T. M. Silva N. F. Vasquez E. C. Meyrelles S. S.. (2011). Hypercholesterolemia promotes early renal dysfunction in apolipoprotein E-deficient mice. Lipids Health Dis. 10, 220. 10.1186/1476-511x-10-220 22117541 PMC3247872

[B6] Baynes J. W. (1991). Role of oxidative stress in development of complications in diabetes. Diabetes 40, 405–412. 10.2337/diab.40.4.405 2010041

[B7] Bouayed J. Bohn T. (2010). Exogenous antioxidants - Double-edged swords in cellular redox state: health beneficial effects at physiologic doses versus deleterious effects at high doses. Oxid. Med. Cell. Longev. 3, 228–237. 10.4161/oxim.3.4.12858 20972369 PMC2952083

[B8] Bradford M. M. (1976). A rapid and sensitive method for the quantitation of microgram quantities of protein utilizing the principle of protein-dye binding. Anal. Biochem. 72, 248–254. 10.1016/0003-2697(76)90527-3 942051

[B9] Candido R. Allen T. J. Lassila M. Cao Z. Thallas V. Cooper M. E.. (2004). Irbesartan but not amlodipine suppresses diabetes-associated atherosclerosis. Circulation 109, 1536–1542. 10.1161/01.CIR.0000124061.78478.94 15023892

[B10] Donate-Correa J. Martín-Núñez E. Muros-de-Fuentes M. Mora-Fernández C. Navarro-González J. F. (2015). Inflammatory cytokines in diabetic nephropathy. J. Diabetes Res. 2015, 948417. 10.1155/2015/948417 25785280 PMC4345080

[B11] Duran-Salgado M. B. Rubio-Guerra A. F. (2014). Diabetic nephropathy and inflammation. World J. Diabetes 5, 393–398. 10.4239/wjd.v5.i3.393 24936261 PMC4058744

[B12] Foggensteiner L. Mulroy S. Firth J. (2001). Management of diabetic nephropathy. J. R. Soc. Med. 94, 210–217. 11385086 10.1177/014107680109400504 PMC1281451

[B13] Galindo P. González-Manzano S. Zarzuelo M. J. Gómez-Guzmán M. Quintela A. M. González-Paramás A. (2012). Different cardiovascular protective effects of quercetin administered orally or intraperitoneally in spontaneously hypertensive rats. Food Funct. 3, 643–650. 10.1039/c2fo10268d 22441211

[B14] Giacco F. Brownlee M. (2010). Oxidative stress and diabetic complications. Circ. Res. 107, 1058–1070. 10.1161/CIRCRESAHA.110.223545 21030723 PMC2996922

[B15] Gomes I. B. Porto M. L. Santos M. C. Campagnaro B. P. Pereira T. M. Meyrelles S. S.. (2014). Renoprotective, anti-oxidative and anti-apoptotic effects of oral low-dose quercetin in the C57BL/6J model of diabetic nephropathy. Lipids Health Dis. 13:184. 10.1186/1476-511X-13-184 25481305 PMC4271322

[B16] Gorin Y. Wauquier F. (2015). Upstream regulators and downstream effectors of NADPH oxidases as novel therapeutic targets for diabetic kidney disease. Mol. Cells 38, 285–296. 10.14348/molcells.2015.0010 25824546 PMC4400302

[B17] Haug M. Awuh J. A. Steigedal M. Frengen Kojen J. Marstad A. Nordrum I. S.. (2013). Dynamics of immune effector mechanisms during infection with *Mycobacterium avium* in C57BL/6 mice. Immunology 140, 232–243. 10.1111/imm.12131 23746054 PMC3784169

[B18] Ishikawa A. Yamashita H. Hiemori M. Inagaki E. Kimoto M. Okamoto M.. (2007). Characterization of inhibitors of postprandial hyperglycemia from the leaves of *Nerium indicum*. J. Nutr. Sci. Vitaminol. (Tokyo) 53, 166–173. 10.3177/jnsv.53.166 17616005

[B19] Jerums G. Panagiotopoulos S. Premaratne E. MacIsaac R. J. (2009). Integrating albuminuria and GFR in the assessment of diabetic nephropathy. Nat. Rev. Nephrol. 5, 397–406. 10.1038/nrneph.2009.91 19556994

[B20] Kanter M. Aktas C. Erboga M. (2012). Protective effects of quercetin against apoptosis and oxidative stress in streptozotocin-induced diabetic rat testis. Food Chem. Toxicol. 50, 719–725. 10.1016/j.fct.2011.11.051 22166789

[B21] Kawabata K. Mukai R. Ishisaka A. (2015). Quercetin and related polyphenols: new insights and implications for their bioactivity and bioavailability. Food Funct. 6, 1399–1417. 10.1039/C4FO01178C 25761771

[B22] Kim J. H. Kang M. J. Choi H. N. Jeong S. M. Lee Y. M. Kim J. I. (2011). Quercetin attenuates fasting and postprandial hyperglycemia in animal models of diabetes mellitus. Nutr. Res. Pract. 5, 107–111. 10.4162/nrp.2011.5.2.107 21556223 PMC3085798

[B23] Kobori M. Masumoto S. Akimoto Y. Takahashi Y. (2009). Dietary quercetin alleviates diabetic symptoms and reduces streptozotocin-induced disturbance of hepatic gene expression in mice. Mol. Nutr. Food Res. 53, 859–868. 10.1002/mnfr.200800310 19496084

[B24] Kwon O. Eck P. Chen S. Corpe C. P. Lee J. H. Kruhlak M.. (2007). Inhibition of the intestinal glucose transporter GLUT2 by flavonoids. FASEB J. 21, 366–377. Epub 2006 Dec 16. Erratum in: *FASEB J*. 21:1942. 10.1096/fj.06-6620com 17172639

[B25] Lara-Guzman O. J. Tabares-Guevara J. H. Leon-Varela Y. M. Álvarez R. M. Roldan M. Sierra J. A.. (2012). Proatherogenic macrophage activities are targeted by the flavonoid quercetin. J. Pharmacol. Exp. Ther. 343, 296–306. 10.1124/jpet.112.196147 22869926

[B26] Lassila M. Seah K. K. Allen T. J. Thallas V. Thomas M. C. Candido R.. (2004). Accelerated nephropathy in diabetic apolipoprotein e-knockout mouse: role of advanced glycation end products. J. Am. Soc. Nephrol. 15, 2125–2138. 10.1097/01.ASN.0000133025.23732.46 15284298

[B27] Like A. A. Rossini A. A. (1976). Streptozotocin-induced pancreatic insulitis: new model of diabetes mellitus. Science 193, 415–417. 10.1126/science.180605 180605

[B28] Lobo V. Patil A. Phatak A. Chandra N. (2010). Free radicals, antioxidants and functional foods: impact on human health. Pharmacogn. Rev. 4, 118–126. 10.4103/0973-7847.70902 22228951 PMC3249911

[B29] Lodi F. Jimenez R. Moreno L. Kroon P. A. Needs P. W. Hughes D. A.. (2009). Glucuronidated and sulfated metabolites of the flavonoid quercetin prevent endothelial dysfunction but lack direct vasorelaxant effects in rat aorta. Atherosclerosis 204, 34–39. 10.1016/j.atherosclerosis.2008.08.007 18801486

[B30] Lv M. Chen Z. Hu G. Li Q. (2015). Therapeutic strategies of diabetic nephropathy: recent progress and future perspectives. Drug Discov. Today 20, 332–346. 10.1016/j.drudis.2014.10.007 25448752

[B31] Machha A. Mustafa M. R. (2005). Chronic treatment with flavonoids prevents endothelial dysfunction in spontaneously hypertensive rat aorta. J. Cardiovasc. Pharmacol. 46, 36–40. 10.1097/01.fjc.0000162769.83324.c1 15965352

[B32] Manach C. Williamson G. Morand C. Scalbert A. Rémésy C. (2005). Bioavailability and bioefficacy of polyphenols in humans. I. Review of 97 bioavailability studies. Am. J. Clin. Nutr. 81(1 Suppl.), 230S–242S. 15640486 10.1093/ajcn/81.1.230S

[B33] Matheus A. S. Tannus L. R. Cobas R. A. Palma C. C. Negrato C. A. Gomes M. B. (2013). Impact of diabetes on cardiovascular disease: an update. Int. J. Hypertens. 2013:653789. 10.1155/2013/653789 23533715 PMC3603160

[B34] Menini S. Iacobini C. Ricci C. Blasetti Fantauzzi C. Pugliese G. (2015). Protection from diabetes-induced atherosclerosis and renal disease by D-carnosine-octylester: effects of early vs late inhibition of advanced glycation end-products in Apoe-null mice. Diabetologia 58, 845–853. 10.1007/s00125-014-3467-6 25471794

[B35] Mogensen C. E. Christensen C. K. Vittinghus E. (1983). The stages in diabetic renal disease. With emphasis on the stage of incipient diabetic nephropathy. Diabetes 32(Suppl. 2), 64–78. 10.2337/diab.32.2.s64 6400670

[B36] Müller O. J. Katus H. A. Backs J. (2012). Macrovascular disease in diabetes: is the mouse a suitable model? Exp. Clin. Endocrinol. Diabetes 120, 194–196. 10.1055/s-0032-1304580 22402944

[B37] Negi B. Kaur R. Dey G. (2013). Protective effects of a novel sea buckthorn wine on oxidative stress and hypercholesterolemia. Food Funct. 4, 240–248. 10.1039/C2FO30125C 23096237

[B38] Oršolic N. Gajski G. Garaj-Vrhovac V. Dikic D. Prskalo Z. Š. Sirovina D. (2011). DNA-protective effects of quercetin or naringenin in alloxan-induced diabetic mice. Eur. J. Pharmacol. 656, 110–118. 10.1016/j.ejphar.2011.01.021 21277296

[B39] Ortega A. Fernández A. Arenas M. I. López-Luna P. Muñóz-Moreno C. Arribas I.. (2013). Outcome of acute renal injury in diabetic mice with experimental endotoxemia: role of hypoxia-inducible factor-1 α. J. Diabetes Res. 2013:254529. 10.1155/2013/254529 23984430 PMC3747493

[B40] Ortega R. García N. (2009). The flavonoid quercetin induces changes in mitochondrial permeability by inhibiting adenine nucleotide translocase. J. Bioenerg. Biomembr. 41, 41–47. 10.1007/s10863-009-9198-6 19296209

[B41] Ozcelik D. Tuncdemir M. Ozturk M. Uzun H. (2011). Evaluation of trace elements and oxidative stress levels in the liver and kidney of streptozotocin-induced experimental diabetic rat model. Gen. Physiol. Biophys. 30, 356–363. 10.4149/gpb_2011_04_356 22131317

[B42] Pendse A. A. Arbones-Mainar J. M. Johnson L. A. Altenburg M. K. Maeda N. (2009). Apolipoprotein E knock-out and knock-in mice: atherosclerosis, metabolic syndrome, and beyond. J. Lipid Res. 50(Suppl.), S178–S182. 10.1194/jlr.R800070-JLR200 19060252 PMC2674752

[B43] Pereira Braga C. Momentti A. C. Barbosa Peixoto F. de Fátima Ferreira Baptista R. dos Santos F. A. Fava F. H.. (2013). Influence of treatment with quercetin on lipid parameters and oxidative stress of pregnant diabetic rats. Can. J. Physiol. Pharmacol. 91, 171–177. 10.1139/cjpp-2012-0173 23458202

[B44] Sanders R. A. Rauscher F. M. Watkins J. B. III. (2001). Effects of quercetin on antioxidant defense in streptozotocin-induced diabetic rats. J. Biochem. Mol. Toxicol. 15, 143–149. 10.1002/jbt.11 11424224

[B45] Schena F. P. Gesualdo L. (2005). Pathogenetic mechanisms of diabetic nephropathy. J. Am. Soc. Nephrol. 16(Suppl. 1), S30–S33. 10.1681/ASN.2004110970 15938030

[B46] Sesink A. L. O'Leary K. A. Hollman P. C. (2001). Quercetin glucuronides but not glucosides are present in human plasma after consumption of quercetin-3-glucoside or quercetin-4'-glucoside. J. Nutr. 131, 1938–1941. 11435510 10.1093/jn/131.7.1938

[B47] Sunarwidhi A. L. Sudarsono S. Nugroho A. E. (2014). Hypoglycemic effect of combination of *Azadirachta indica* A. Juss. and *Gynura procumbens* (Lour.) Merr. Ethanolic extracts standardized by rutin and quercetin in alloxan-induced hyperglycemic rats. Adv. Pharm. Bull. 4, 613–618. 10.5681/apb.2014.090 25671197 PMC4312413

[B48] Ulasova E. Perez J. Hill B. G. Bradley W. E. Garber D. W. Landar A.. (2013). Quercetin prevents left ventricular hypertrophy in the Apo E knockout mouse. Redox Biol. 1, 381–386. 10.1016/j.redox.2013.07.001 24024175 PMC3757709

[B49] Valensi P. Le Devehat C. Richard J. L. Farez C. Khodabandehlou T. Rosenbloom R. A.. (2005). A multicenter, double-blind, safety study of QR-333 for the treatment of symptomatic diabetic peripheral neuropathy. A preliminary report. J. Diabetes Complications 19, 247–253. 10.1016/j.jdiacomp.2005.05.011 16112498

[B50] Vedantham S. Noh H. Ananthakrishnan R. Son N. Hallam K. Hu Y.. (2011). Human aldose reductase expression accelerates atherosclerosis in diabetic apolipoprotein E-/- mice. Arterioscler. Thromb. Vasc. Biol. 31, 1805–1813. 10.1161/ATVBAHA.111.226902 21636809 PMC3278231

[B51] Vessal M. Hemmati M. Vasei M. (2003). Antidiabetic effects of quercetin in streptozocin-induced diabetic rats. Comp. Biochem. Physiol. C Toxicol. Pharmacol. 135C, 357–364. 10.1016/S1532-0456(03)00140-6 12927910

[B52] Wang C. Pan Y. Zhang Q. Y. Wang F. M. Kong L. D. (2012). Quercetin and allopurinol ameliorate kidney injury in STZ-treated rats with regulation of renal NLRP3 inflammasome activation and lipid accumulation. PLoS ONE 7:e38285. 10.1371/journal.pone.0038285 22701621 PMC3372527

[B53] Wen M. Segerer S. Dantas M. Brown P. A. Hudkins K. L. Goodpaster T. (2002). Renal injury in apolipoprotein E-deficient mice. Lab. Invest. 82, 999–1006. 10.1097/01.LAB.0000022222.03120.D4 12177238

[B54] Wright E. Jr. Scism-Bacon J. L. Glass L. C. (2006). Oxidative stress in type 2 diabetes:the role of fasting and postprandial glycaemia. Int. J. Clin. Pract. 60, 308–314. 10.1111/j.1368-5031.2006.00825.x 16494646 PMC1448694

[B55] Wu J. Yan L. J. (2015). Streptozotocin-induced type 1 diabetes in rodents as a model for studying mitochondrial mechanisms of diabetic β cell glucotoxicity. Diabetes Metab. Syndr. Obes. 8, 181–188. 10.2147/DMSO.S82272 25897251 PMC4396517

[B56] Wu K. K. Huan Y. (2007). Diabetic atherosclerosis mouse models. Atherosclerosis 191, 241–249. 10.1016/j.atherosclerosis.2006.08.030 16979174

[B57] Xu M. Hu J. Zhao W. Gao X. Jiang C. Liu K.. (2014). Quercetin differently regulates insulin-mediated glucose transporter 4 translocation under basal and inflammatory conditions in adipocytes. Mol. Nutr. Food Res. 58, 931–941. 10.1002/mnfr.201300510 24343960

[B58] Xu S. Jiang B. Maitland K. A. Bayat H. Gu J. Nadler J. L.. (2006). The thromboxane receptor antagonist S18886 attenuates renal oxidant stress and proteinuria in diabetic apolipoprotein E-deficient mice. Diabetes 55, 110–119. 10.2337/diabetes.55.01.06.db05-0831 16380483

[B59] Youl E. Bardy G. Magous R. Cros G. Sejalon F. Virsolvy A.. (2010). Quercetin potentiates insulin secretion and protects INS-1 pancreatic β-cells against oxidative damage via the ERK1/2 pathway. Br. J. Pharmacol. 161, 799–814. 10.1111/j.1476-5381.2010.00910.x 20860660 PMC2992896

[B60] Zhao L. R. Du Y. J. Chen L. Liu Z. G. Pan Y. H. Liu J. F. (2014). Quercetin protects against high glucose-induced damage in bone marrow-derived endothelial progenitor cells. Int. J. Mol. Med. 34, 1025–1031. 10.3892/ijmm.2014.1852 25197782

